# Travelers’ diarrhea: update on the incidence, etiology and risk in military and similar populations – 1990-2005 versus 2005–2015, does a decade make a difference?

**DOI:** 10.1186/s40794-018-0077-1

**Published:** 2019-01-15

**Authors:** Scott Olson, Alexis Hall, Mark S. Riddle, Chad K. Porter

**Affiliations:** 1 Enteric Disease Department, Naval Medical Research Center, 503 Robert Grant Avenue, Silver Spring, MD 20910 USA; 2 Uniformed Services University of the Health Sciences, Bethesda, MD USA

**Keywords:** Travelers’ diarrhea, Long term traveler, Enterotoxigenic *E. coli* (ETEC), Enteroaggregative *E. coli* (EAEC), Campylobacter

## Abstract

**Background:**

Travelers’ diarrhea remains a prevalent illness impacting individuals visiting developing countries, however most studies have focused on this disease in the context of short term travel. This study aims to determine the regional estimates of travelers’ diarrhea incidence, pathogen-specific prevalence, and describe the morbidity associated with diarrheal disease among deployed military personnel and similar long term travelers.

**Methods:**

We updated a prior systematic review to include publications between January 1990 and June 2015. Point estimates and confidence intervals of travelers’ diarrhea and pathogen prevalence were combined in a random effects model and assessed for heterogeneity. Eighty-two studies were included in the analysis, including 29 new studies since the prior systematic review.

**Results:**

Military personnel were evaluated in 69% of studies and non-military long term travelers in 34%, with a median duration of travel of 4.9 months, and travel predominantly to the Middle East, Southeast Asia, and Latin America and the Caribbean. Sixty-two percent of tested cases were due to bacterial pathogens, with enterotoxigenic *E. coli* (ETEC), enteroaggregative *E. coli* (EAEC), and *Campylobacter* predominating, and significant regional variability. The incidence of TD from studies with longitudinal data was 36.3 cases per 100 person-months, with the highest rates in Southeast Asia, Latin America and the Caribbean, and the Middle East, with higher estimates from those studies using self-reporting of disease. Morbidity remained significant, with 21% being incapacitated or placed sick in quarters (SIQ) by their illness, 15% requiring intravenous fluids, and 3% requiring hospitalization.

**Conclusions:**

In comparison to results from the prior systematic review, there were no significant differences in incidence, pathogen prevalence, or morbidity; however there was a trend toward improved care-seeking by sick individuals.

**Electronic supplementary material:**

The online version of this article (10.1186/s40794-018-0077-1) contains supplementary material, which is available to authorized users.

## Introduction

In the United States foodborne disease affects 1 in 6 individuals per year, and while the majority (80%) of cases are due to unidentified causes, among diagnosed etiologies in this setting, viruses predominate [1]. While most cases are self-limited and do not require targeted therapy, enteric illnesses account for an estimated annual cost of $93.2 billion [1]. In contrast, among travelers to developing countries, the rate of acute enteric disease has been historically much higher, with an estimated attack rate of 29% per month [2]. In addition, bacteria predominate as the cause of acute illness, with diarrheagenic *E. coli*, *Campylobacter spp.*, and *Shigella spp.* representing the most commonly isolated pathogens [3–5]. And while rates of travelers’ diarrhea (TD) among short-term travelers (less than one month) may be decreasing [6], we previously reported unchanged rates of disease among long-term travelers [2].

Deployed military personnel represent a unique subset of travelers, among whom TD historically has caused significant morbidity and mortality. Throughout recorded history, the importance of infectious enteric disease has been noted, with mentions of diarrheal illness among troops as early as the Peloponnesian War [7]. In American history, acute enteric illness has been noted to hinder military operations in every major war, from the Civil War through the Vietnam War [7]. More recently, data from Operation Iraqi Freedom (OIF) and Operation Enduring Freedom (OEF) highlight that acute diarrheal illness has remained the most common non-combat disease among deployed US personnel, with incidence as high as 45 episodes per 100 person-months [8–10].

The risks for developing acute TD are similar between military and non-military long-term travelers, with first time travel or deployment, travel or deployment to developing nations, younger age, and lack of dietary discretion being common risk factors [5, 6, 11, 12]. Among both military and non-military travelers, individuals afflicted by TD during their travel are often precluded from participation in daily activities and work. This scenario in the military can result in potentially high numbers of lost duty days across an entire deployed population, and close living conditions can lead to large outbreaks [10, 13–15].

Our 2006 systematic review highlighted the incidence, etiology, and impact of travelers’ diarrhea among US military personnel and similar travelers [2]. Since that time major changes to the pattern and tempo of military deployment and multiple new studies of TD in this population necessitate an update to the prior systematic review to include studies published since 2005 and better describe regional estimates of diarrheal disease incidence, pathogen-specific prevalence, and management options and morbidity among long-term travelers, including deployed US military personnel and similar populations. In updating the prior systematic review, we demonstrate how diarrheal illness has changed among long term travelers in the past decade, so that in both the civilian and military populations providers can better manage this prevalent disease using the most current data.

## Methods

We performed a systematic review of the scientific literature published between July 1, 2005 and June 30, 2015, based on accepted principles of methodological design [16, 17], including eligibility criteria for available evidence, standardized data abstraction, critical appraisal of the evidence, and standardized methods of data analysis. These data were then combined with data from our previous 2006 study.

### Search strategy and study selection

We conducted our review initially by performing a comprehensive search of electronic bibliographic databases (including MEDLINE, EMBASE, CINAHL, and the Cochrane Library). Additionally manual searches of bibliographies of identified studies, technical reports, and doctoral dissertations were performed. All searches were performed by pairing keyword(s) [travelers’ diarrhea or diarrhea] with each of the following terms eliminating duplicates: epidemiology, etiology, military, Peace Corps, expatriate, incidence, burden, morbidity, and treatment. In addition, MEDLINE searches were conducted using major MeSH headings (medical subject headings) determined from articles known to be eligible. One reviewer screened all publications and reports for possible inclusion based on eligibility criteria. If eligibility could not be determined from title and/or abstract alone, the full-text was retrieved and evaluated. Data from retrieved articles, technical reports, and doctoral dissertations were abstracted based on inclusion and exclusion criteria then verified. All review articles were obtained for hand searching bibliographies to identify further potential articles for inclusion.

Original research in the form of observational cohorts, surveys, database analyses, or clinical trials published in English were considered for inclusion. Publications classified as review, in non-English language, editorials, case-reports, or irrelevant were excluded, and reasons for exclusion were recorded. Studies on US military and other similar or long-term travel populations were reviewed for inclusion criteria. Similar traveler populations were defined as expatriates of a developed country traveling or living abroad in a developing country. Long-term travel was defined as travel for ≥1 month. All studies involving US military, regardless of travel duration, were considered for inclusion. Studies involving tourists and short-term (< 1 month of travel abroad) business travelers were excluded. Studies with duplicate study populations were also excluded. Studies were categorized to geographic regions of sub-Saharan Africa, Latin America and the Caribbean, Southeast Asia, and the Middle East based on reported geographic regions of travel. Only publications reporting information for variables of interest to this study were included.

### Data abstraction and validation

Using the same pre-tested, standardized abstraction form (included in Additional file 1) that was used for our prior systematic review, we abstracted data from retrieved articles and reports. Bibliographic information, study design description, study years, geographic location, and population characteristics (e.g., follow-up period, case definition, etc.) necessary to answer key questions and to evaluate heterogeneity of studies were included in the data abstraction form [2].

The primary outcomes of interest included quantitative and qualitative measures of etiology and incidence of TD and its associated treatment and morbidity. Pathogen prevalence was abstracted as a percentage of total cases reported, along with the study denominator that was used to calculate the prevalence. Because of the difficulty in determining the causality of disease when multiple pathogens are isolated, pathogen prevalence was reported as the number of cases infected with a specific agent inclusive of cases with multiple organisms. The total number of diarrheal cases was abstracted along with the size of the study population and study period used to compute diarrheal prevalence/incidence for each separate study. Incidence was abstracted as an event number, with person-time as the denominator when available. In prophylaxis trials, estimates were only abstracted for the placebo group. Estimates were stratified based on self-reporting, clinic-based case series, or disease and non-battle injury (DNBI) reports. Data were then combined with the 2006 review data.

### Data analysis

Pathogen prevalence and incidence were stratified by region as geographic differences as previously described [18, 19]. Because of known variations in study design, methodologies, population characteristics, and other factors, heterogeneity of prevalence and incidence estimates across studies was expected and assessed graphically with Forest plots and statistically through the use of heterogeneity statistics and non-parametric methods. For the purpose of summary, point estimates and 95% confidence intervals were calculated using a random effects model with methodology developed by DerSimonian and Laird [20].

In the case of parameters where only a few studies were found (e.g., probabilities and outcomes associated diarrhea and treatment), a median and range of estimates are reported. Publication bias was not assessed, because the concern of non-published findings caused by negative studies or disappointing results was considered to be minimal, given the objective of summarizing pathogen prevalence and disease incidence.

All analyses were conducted using Stata V13 (StataCorp, College Station, TX).

## Results

**Fig. 1:**
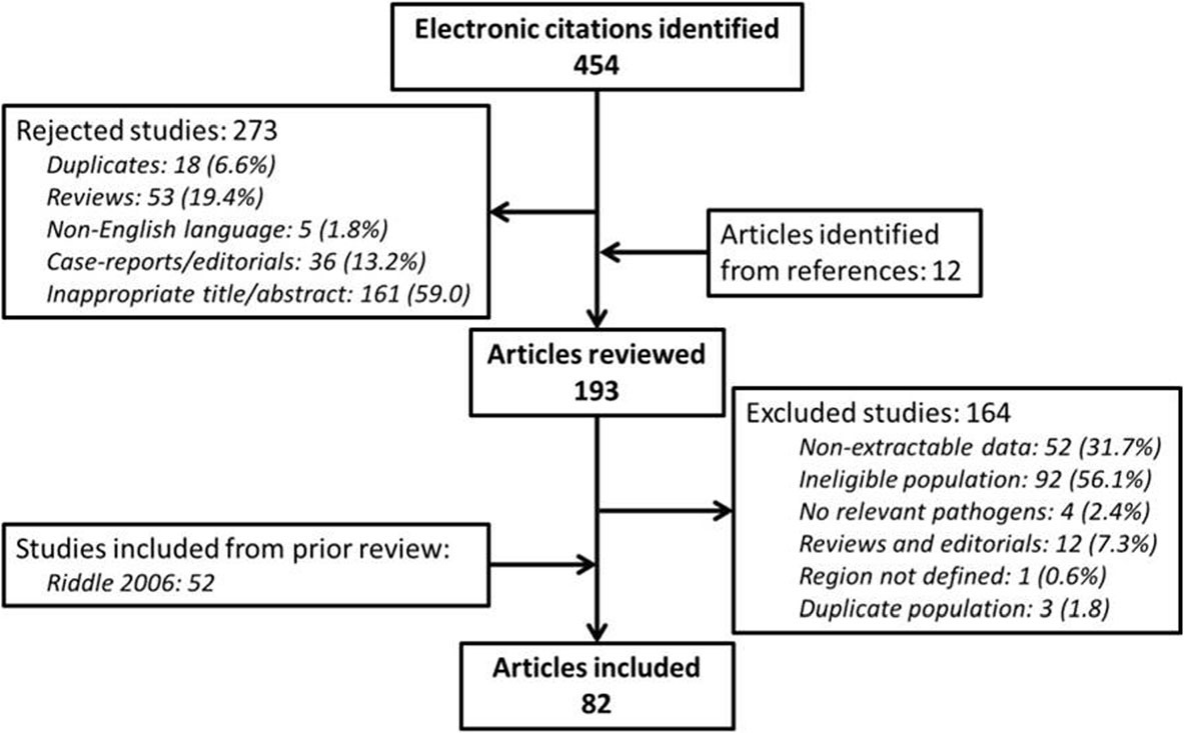
Flow diagram of study selection for inclusion in the updated systematic review. We identified 454 candidate studies for inclusion since the last systematic review by query of electronic bibliographic databases. After review of titles and abstracts, 193 studies were identified for full review, with 164 studies excluded. Thirty studies were identified for inclusion in the final systematic review, with a total of 82 studies when those from the prior review were included

Our initial search of the literature identified 454 eligible articles. Of those studies 273 were rejected on review of abstracts due to being duplicate studies (*n* = 18), reviews (*n* = 53), case reports or editorials (*n* = 36), or finding inappropriate titles or abstracts (*n* = 161), with an additional 164 studies excluded after full review due to ineligible populations (*n* = 92), non-extractable data (*n* = 52), or being reviews or editorials (*n* = 12). Twelve additional studies were identified from the references of retrieved articles. Following evaluation for suitability, 29 articles were identified for final inclusion, abstracted, scored for quality, and combined with data from the precursor review (*n* = 52). Results from an additional unpublished study were also identified and included following review of the available draft manuscript, yielding a total of 82 included studies in the updated systematic review [9, 14, 18, 21–96]. The study selection process is detailed further in Fig. 1.

### Study characteristics

As shown in Table 1, the median sample size of the studies included in this systematic review was 240 travelers (interquartile range [IQR] 114, 1529). The travel population identified in these studies was primarily composed of deployed US military personnel (54 studies, 69%), with non-US military populations evaluated in 28 studies (34%). The median duration of travel to developing nations was 4.9 months (IQR 1, 5.7), with travel predominantly to the Middle East/North Africa region (52% of travelers), followed by S.E. Asia and Latin America/Caribbean (each 14%) and Sub-Saharan Africa and “Multiple regions” (each 10%). The included studies ranged in study design, with descriptive studies (*n* = 25, 30%), clinical trials (*n* = 16, 19%), and cohort studies (*n* = 12, 15%) representing the most common designs. A majority of studies (*n* = 59, 72%) included the standard definition of travelers’ diarrhea (at least three loose stools in a 24-h period or at least two loose-stools in a 24-h period with associated symptoms). The median year of all studies was 1999.

**Table 1:** Studies included in current systematic review of diarrheal illness among long term travelers since 2006

Reference	First Author	Year of Pub	Study Years	Study Design	Country	Population	Population Size	Travel Duration	Percent Male	Age
	Middle East									
[24]	Armstrong, AW	2010	2007–2008	Clinical Trial	Turkey	US Military	100	0.5	88	36
[28]	Brown, J	2009	2004	Cross-sectional	Multiple	US Military	3933		89	25
[45]	Hillel, O	2005	2003	Cross-sectional	India	Backpackers	114	5	43	26.6
[55]	Letizia, A	2014	2005–2006	Clinical Trial	Turkey	US Military	109	12	86	
[58]	Monteville, MR	2006	2004	Other	Iraq; Afghanistan	US Military	194	4.8	92	32
[58]	Monteville, MR	2006	2004	Other	Iraq; Afghanistan	US Military	28,322			
[70]	Porter, C	2010	2002	Mixed design	Turkey	US Military	202	4	89	34
[68]	Porter, C	2011	1999–2007	Case-control	Iraq; Afghanistan	US Military	31,866		54.5	28.5
[69]	Porter, C	2011	2008–2009	Case-control	Afghanistan, Iraq	US Military	535	6	56.6	26
[16]	Putnam, S	2006	2003–2004	Cross-sectional	Iraq; Afghanistan	US Military	10,833	11	89.6	26
[9]	Riddle, MS	2008	2006–2007	Cross-sectional	Iraq; Afghanistan; Kuwait	US Military	3374	5.7	84.5	26
[72]	Riddle, MS	2011	2004–2005	Mixed design	Egypt	US Military	211	5.7		31
[75]	Sanders, JW	2007	2003–2004	Clinical Trial	Turkey	US Military	207	8	65	31
[96]	Sanders, JW	2005 (conf)	2004	Cross-sectional	Iraq	US Military	537	3.7	98.1	23
[79]	Sebeny, PJ	2012	2009	Mixed design	Egypt	US Military	1529	0.7	81	34
[89]	Trivedi, KH	2011	2004–2005	Cohort	Egypt; Turkey	US Military	120	3.7	74	34
	SE Asia									
[66]	Piyaphanee, W	2011	2009	Cross-sectional	Thailand	Backpackers	404	1	59.7	26
[88]	Tribble, DR	2007	2000–2001	Clinical Trial	Thailand	US Military	156		89	26
[87]	Tribble, DR	2008	2000–2001	Mixed design	Thailand	US Military	182		90	26
[93]	Velasco, J	2015	2014	Other - Surveillance	Philippines	US Military	15	0.5	100	29
	Latin America									
[94]	Ajami, N	2014	2004	Descriptive study	Mexico	Students	75	1	46	26
[52]	Kasper, MR	2012	2011	Mixed design	El Salvador	US Military	241	0.5	83.8	27
[71]	Reaves, EJ	2012	2012	Mixed design	Peru	US Military	101			
	Latin America and Caribbean									
[95]	Chern, A	2016	2011	Mixed design	Multiple	US Military / Civilian	3156	5	64.6	31
	Africa									
[39]	Frickmann, H	2015	2013–2014	Surveillance	Mali	non-US Military	51	8.5		
[56]	Marimoutou, C	2011	2007–2008	Cohort	Chad	non-US Military	278	5		
[67]	Pommier de Santi, V	2011	2007–2008	Mixed design	Chad	non-US Military	240	5	91.7	27
	Multiple									
[30]	Chen, L	2009	1996–2008	Descriptive study		Peace Corps; Expatriate	4039	12	57.2	
[83]	Stermer, E	2006	2004	Cross-sectional		Non-military Travelers	405	1	53.3	30.8
[90]	Tuteja AK	2008		Cohort		Missionaries (mainly)	83	16	82	21

This table lists all studies included in the current systematic review since the last review in 2006. Thirty studies were included for analysis, bringing the total number of studies in this systematic review to 82. The author, year of publication, study years, type of study design, countries included, type of travel population evaluate, population size, median travel duration, percent male, and median age are included for each included study, where available. The studies are listed by region of travel

### Pathogen prevalence

**Fig. 2:**
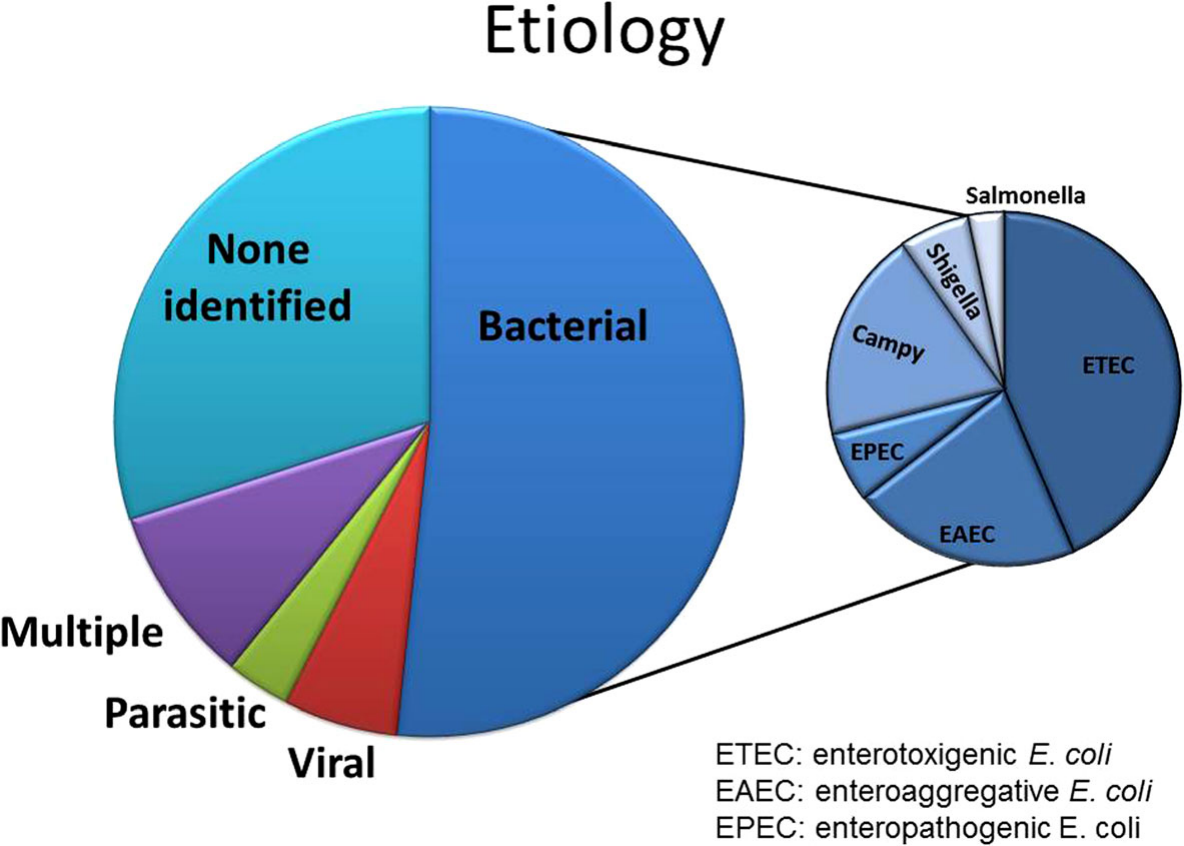
Pathogen prevalence of diarrheal illness among long term travelers, 1990–2015. Of those cases for which diagnostic testing was performed, 62% were positive for bacterial pathogens, with viruses (7%) and parasites (4%) being detected at lower rates. ETEC was the most commonly isolated bacterial etiology (44%), followed by EAEC (21%) and *Campylobacter* spp. (19%). Multiple pathogens were detected in 11% of tested cases and no identified pathogens in 36%

Fifty-two studies (63%) reported etiologies of acute TD, with the majority of cases (64% of 11,613 total) having an identified pathogen recovered. Bacterial pathogens were identified in 62% of cases where testing was performed (10,552 tested), with viral (7% of 5443 tested) and parasitic (4% of 5905 tested) pathogens being detected at much lower frequencies (Fig. 2). Recovery of multiple pathogens was found in 11% of tested TD cases, and no pathogens were detected in 36% of cases. There was marked heterogeneity among studies estimating prevalence for individual pathogens across all regions (χ^2^ heterogeneity statistic, *P* < 0.001); pathogens included in the analysis were those listed in Table 2, and regions included were defined as SE Asia, Africa, Middle East, and Latin America/Caribbean. Among identified bacterial causes, enterotoxigenic *E. coli* (ETEC, 44% of bacterial cases), enteroaggregative *E. coli* (EAEC, 21%), and *Campylobacter* spp. (19%) were most commonly reported. Other bacterial pathogens reported at lower rates include enteropathogenic *E. coli* (EPEC, 6% of bacterial cases), *Shigella* (6%), and *Salmonella* spp. (3%). There were no significant differences in prevalence of various pathogens between studies before and after 2006 (Table 2).

**Table 2:** Comparison of pathogen prevalence between prior review and update

Pathogen	n	Median	IQR
ETEC			
- Prior study	34	20.5	11.8, 32.0
- 2006–2015	13	22.0	12.2, 40.6
Campylobacter			
- Prior study	32	4.0	0.9, 12.5
- 2006–2015	14	7.2	1.6, 18.1
Shigella			
- Prior study	34	3.5	1.0, 9.0
- 2006–2015	13	6.1	1.0, 8.6
Salmonella			
- Prior study	32	2.5	1.0, 6.5
- 2006–2015	12	1.1	0.0, 3.1
EAEC			
- Prior study	14	8.5	2.8, 11.5
- 2006–2015	6	9.2	0.0, 11.0
No etiology identified			
- Prior study	32	50.0	35.5, 56.3
- 2006–2015	9	39.0	20.6, 46.5
Multiple pathogens			
- Prior study	17	9.0	6.0, 16.0
- 2006–2015	12	11.0	6.1, 22.6

The prevalence of specific bacterial pathogens identified by testing. Prevalence was compared between the prior systematic review and studies performed since that time (2006–2015). The number of studies in each subgroup (n) is included. Comparison of median values between groups was analyzed using Wilcoxon signed-rank testing

**Fig. 3:**
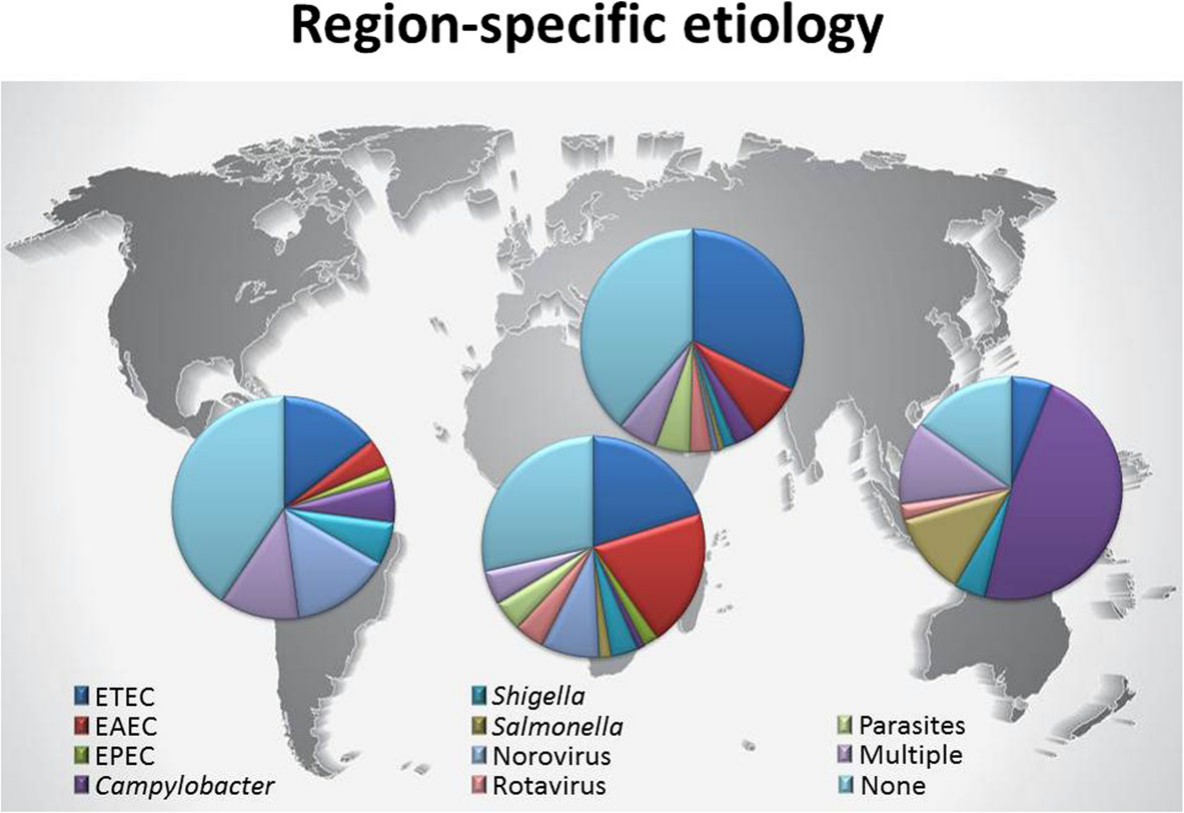
Region specific pathogen prevalence of diarrheal illness among long term travelers, 1990–2015. We identified variability of pathogen prevalence across studies from different regions. In Latin America/Caribbean, Africa, and the Middle East, the most common identified pathogen was ETEC, while in SE Asia *Campylobacter* spp. predominated

There was notable variability in the prevalence of specific pathogens between regions. ETEC and EAEC were predominant pathogens in Africa (36% each of total identified causes) and ETEC in the Middle East (39%), while *Campylobacter* (63%) and *Salmonella* (16%) were the most commonly isolated pathogens in SE Asia. Although viruses were less frequently identified pathogens, norovirus represented a higher proportion of cases in Latin America/Caribbean (14%) than other regions (Fig. 3). The rates of multiple pathogen recovery ranged from 7% of cases in the Middle East to 16% in SE Asia. Across regions, there was also variability in the rates of successful pathogen identification, with no identified pathogen found in 20% of tested subjects in SE Asia compared to 51% in Africa.

Pathogen prevalence also varied depending on the type of traveler assessed. In US military populations (5520 cases tested), ETEC (28% of identified pathogens), *Campylobacter* (15%), and EAEC (9%) were most commonly detected, with multiple pathogens detected in 9% of cases and no identified pathogen in 35%. Among non-US military populations (6093 cases tested) EAEC (36% of identified pathogens), ETEC (19%), and norovirus (15%) predominated, with multiple pathogens identified in 16% of cases and no identified pathogen in 51%.

### Incidence

**Fig. 4:**
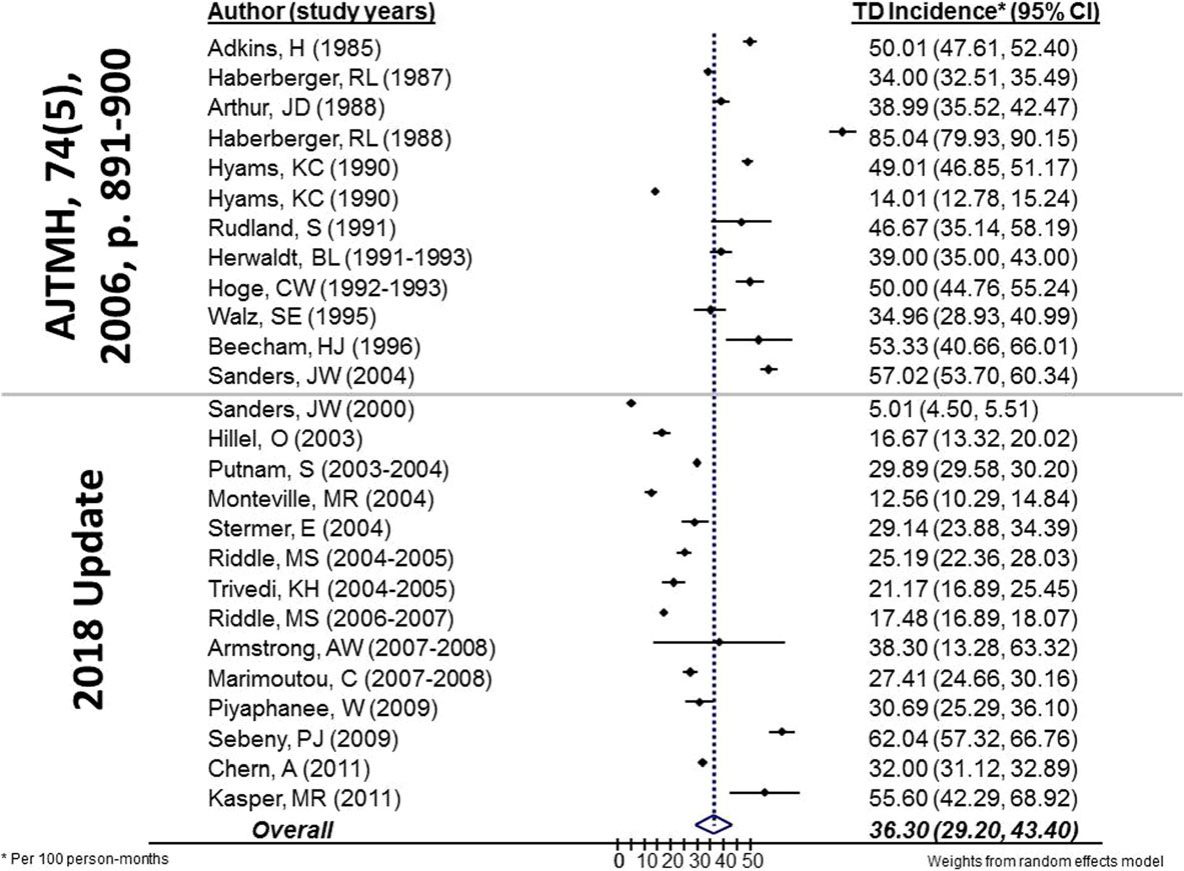
Incidence of diarrheal illness among long-term travelers, 1990–2016. We found an estimated overall pooled incidence of TD among long-term travelers of 36.3 cases per 100 person-months. Studies included in the prior systematic review (AJTMH, 64(5), 2006) are listed at the top of the forest plot, with studies since that time included at the bottom of the figure (2018 Update). Weights calculated by random effects model

Fifty-three studies (64%) reported incidence of TD among 65,555 long-term travelers. As with pathogen prevalence there was considerable heterogeneity between studies used to estimate diarrhea incidence (χ^2^ heterogeneity statistic, *P* < 0.001). We estimated a pooled incidence of TD among long-term travelers of 36.3 cases per 100 person-months (95% confidence interval [CI] 29.2–43.4, 26 included studies) (Fig. 4). The incidence of TD appeared to be greatest within the first month of travel (48.9 per 100 person-months before 1 month vs. 31.3 after 1 month; *p* < 0.001). There was heterogeneity in TD incidence across regions, with the highest incidence experienced by travelers to SE Asia (41 cases per 100 person-months, 5 studies), Latin America/Caribbean (39.4, 3 studies), and the Middle East (35.2, 14 studies) (*p* < 0.001). Lower incidence of TD was found in S. Asia (33.3, 2 studies) and Africa (27.4, 1 study). Because it represented the largest regional dataset, we evaluated the subset of long-term travelers to the Middle East for any temporal trends in incidence and found TD had not changed significantly from 2006 (29.4, IQR 13.6, 45.2; 14 studies) to 2015 (27.2, IQR 20, 36.2; 12 studies).

Among the 53 studies, twenty-seven studies estimated TD incidence by clinic-based reporting of subjects presenting to care for acute illness. The overall pooled estimate based on these studies was 7.7 cases per 100 person-months (95% CI 6.5–9.0), with no significant differences noted between US military populations (7.6, 95% CI 6.2–8.9) and other travelers (7.4, 95% CI 5.5–9.3). Twenty-six studies estimated incidence by either self-reporting of symptoms or cohort study design with an overall pooled estimate of 36.3 cases per 100 person-months (95% CI 39.9–41.7), and no significant differences between US military (37.5, 95% CI 30.7–44.4) and other travelers (33.2, 95% CI 27.7–38.8).

The rate of seeking care appeared to have increased since the 2006 review. Within the subset of studies from 1990 to 2005 that reported care-seeking behaviors, only 16% of sick individuals (95% CI 12–21%, 7 studies) sought medical care, while from 2006 to 2015 38% sought care (95% CI 32–45%, 7 studies; *p* = 0.012). The cumulative rates of care-seeking across all included studies was 25% (95% CI 22–29%).

### Morbidity

Thirty-five studies (43%) had extractable information that described the parameters of disease morbidity, including disease severity and treatment outcomes. A total of 21% of subjects reported being sick-in-quarters (SIQ) or incapacitated by their illness, 15% required IV fluids, and 3% required inpatient hospital admission for treatment (Table 3). Additionally there appeared to be a higher rate of travelers placed SIQ or incapacitated by their acute illness when *Shigella* or *Campylobacter* spp. were isolated compared to other bacterial etiologies, though this was not a statistically significant finding. Nine studies reported the probability of treatment failure among travelers reporting for care, with a median rate of failure of 9%. Thirty-six included studies (44%) reported the morbidity of treated and/or untreated disease. For travelers with TD who did not seek care or receive treatment, the median duration of symptoms was 3.0 (IQR 2.4–3.5) days. Those who did seek care reported a median of 1.4 days of symptoms before presenting to medical care, and 1.5 days of symptoms after treatment. Of those who presented to care, 85% received an anti-motility agent (loperamide), while 57% received antibiotic medications. The time to last unformed stool was shorter for travelers administered antibiotics plus loperamide versus those receiving antibiotics alone (9.6 vs. 21.8 h), though this difference was not statistically significant (*p* = 0.2).

**Table 3:** TD associated outcomes (‘90 – ‘15)

Outcome	All years			1990–2005			2006–2015		
	Median	IQR	Min, Max	Median	IQR	Min, Max	Median	IQR	Min, Max
Pre-treatment symptoms, days	1.4	1.1, 2.0	0.3, 4.0	1.4	1.1, 1.7	0.3, 4.1	2.0	1.0, 3.0	1.0, 3.3
Post-treatment symptoms, days	1.5	1.1, 1.9	0.6, 2.2	1.4	0.8, 1.8	0.6, 2.2	1.8	–	1.5, 2.0
- TLUS (abx + loperamide), hrs	9.6	8.2, 11.5	8.2, 11.5	11.5	–	–	8.9	–	8.2, 9.6
- TLUS (abx – loperamide), hrs	21.8	11.0, 43.2	11.0, 43.2	16.4	–	11.0, 21.8	43.2	–	–
Prob. Receiving Abx, %*	57.0	19.0, 91.0	3.0, 100.0	79.0	–	–	44.0	15.0, 96.0	3.0, 100.0
Prob. Receiving loperamide, %*	85.0	27.0, 88.0	17.0, 90.0	–	–	–	85.0	27.0, 88.0	17.0, 90.0
Prob. Receiving IV fluids, %*	15.0	6.0, 19.0	0.0, 33.0	12.0	0.0, 15.0	0.0, 15.0	17.0	6.0, 23.0	5.0, 33.0
Prob. SIQ or incapacitation, %*	21.0	13.0, 34.0	3.0, 55.0	16.0	5.0, 46.0	3.0, 55.0	23.0	13.0, 34.0	4.0, 39.0
Prob. Hospital admission, %	3.0	1.0, 13.0	0.0, 17.0	11.0	–	10.0, 13.0	2.0	1.0, 10.0	0.0, 17.0
Symptom duration in those not seeking treatment, days	3.0	2.4.0, 3.5.0	1.0, 4.3.0	3.0.0	2.5, 3.8	2.1, 4.3	2.9	1.8, 3.6	1.0, 3.8

TLUS – time to last unformed stool

SIQ – sick in quarters

*among those seeking care

Outcomes of interest, including those reflecting the morbidity of diarrheal illness, were compared. Outcomes were compared from all years, from studies included in the prior systematic review (1990–2005), and from studies performed since that time (2006–2015)

## Discussion

This updated review reveals that travelers’ diarrhea remains a major medical concern among deployed US military and other long-term travelers, with a pooled incidence of over 30 cases per 100 person-months, and the highest incidence in the first month of travel. In addition to this high rate of illness, we found no notable change in incidence over the last 25 years of available data. This finding differs from previous reviews of TD among all travelers that suggest a decrease in incidence over the past two decades [6]. Since one could assume that eating local fare or consuming improperly treated water while traveling would drive the continued high rate of diarrheal disease while traveling, the restricted access of military personnel deployed to active theaters (Middle East) should preclude eating food or drinking water locally acquired. Our finding that the rate of TD among US military personnel is similar to other long term travelers would contradict this conclusion, suggesting that off base exposures are ubiquitous and food and water control is difficult [10]. Given that the incidence of disease remains high despite pre-travel education and counseling, other preventative measures, namely development of successful vaccines against the most common pathogens, should remain a focus in ongoing research. In addition to the continued high rates of disease, we found that incidence is much higher when studies use self-reporting, reflecting that studies relying on active surveillance (clinic or DNBI-based) likely underestimate the true incidence of TD [97]. And while our last systematic review found that there was no appreciable difference in incidence across different regions, in this update we found that incidence of disease was higher in SE Asia than all other included regions. Prior reviews have also noted regional variability, with higher rates of TD in SE Asia, South Asia, Northern and Sub-Saharan Africa, and the Middle East compared to other travel destinations, with an overall trend of decreasing rates of TD in South America and East and Southeast Asia [4, 6, 12, 98].

We found that in those studies where evaluation for specific infectious etiologies was performed, a majority of subjects had at least one pathogen identified, a finding consistent with other reviews that have reported pathogen recovery ranging from 50 to 94% of TD cases [3, 4, 6]. Consistent with estimates from our prior systematic review, diarrheagenic *E. coli* (particularly ETEC and EAEC) and *Campylobacter* remained the most common pathogens causing TD in aggregate. As was demonstrated in the prior review, we again note significant variability of enteropathogens across geographic regions, with SE Asia experiencing the highest prevalence of *Campylobacter* and *Salmonella* infections, and higher rates of norovirus recovery in South America. Our findings correspond with an epidemiologic survey by Shah et al., in which ETEC was the predominant enteropathogen across all regions, found in 30% of all subjects studied, followed by EAEC, with higher prevalence of norovirus and rotavirus in Latin America and Africa, and *Campylobacter* and *Salmonella* in Southeast Asia [3].

In addition to the variability of specific etiologies, we also found that the rates of successful isolation of pathogens varied by region, ranging from only about 50% in Africa up to almost 80% in SE Asia. This finding likely reflects the heterogeneity of study designs. And while improvements in storage and processing (including culture-independent detection methods) have occurred in the last two decades, we did not note any apparent improvement in pathogen recovery over this time period. Shah et al. noted that while use of PCR diagnostics resulted in a higher rate of detection of diarrheagenic *E. coli* species not detected on routine culture, this finding has not been replicated in larger epidemiologic studies [3]. We anticipate studies utilizing molecular diagnostics for pathogen identification will reveal whether viral pathogens such as norovirus represent a large proportion of the unidentified cases. While recent studies have found norovirus to be the second leading cause of TD after ETEC, analysis of samples in the Global Enteric Multicenter Study (GEMS) comparing culture-based and culture-independent molecular diagnostic methods found higher estimates of incidence of disease from ST-ETEC and *Shigella* with PCR-based diagnostics, but similar estimates of viral (Norovirus) and parasitic (*Cryptosporidium*) pathogens [99–101]. As the GEMS study aimed to characterize diarrheal illness among children less than 5 years old in developing countries, it is possible that pathogen prevalence will inherently differ from our findings, largely due to the difference between local children and international travelers in epidemiologic exposures within the same geographic location.

Based on reported duration of symptoms from subjects not seeking care, we found that the total duration of symptoms of uncomplicated disease is around three days. Interestingly, we found that duration of symptoms among individuals seeking care was similar (1.4 days before treatment and 1.5 days after). It is likely that as TD is routinely a self-limited disease, treatment may mitigate severity but not duration of symptoms. While we did not find a difference in duration of symptoms between treated and untreated travelers, within the group of travelers who received treatment, data on time to last unformed stool (TLUS) demonstrates that subjects administered a combination of loperamide plus antibiotic reported more rapid recovery than those using only antibiotics, with a difference of almost 20 h. In contrast to our findings, a Cochrane review of TD found quicker resolution of symptoms with antibiotic therapy [102]. In addition a recent study of single-dose antibiotic treatment of TD among deployed military personnel found that treated individuals experienced TLUS of around 12 h compared to greater than 72 h in historically untreated controls [103].

Despite this relative short duration of symptoms, there remains a significant burden of morbidity due to more severe cases, with a high rate of individuals being incapacitated as a result of their illness or requiring IV rehydration. Our results are consistent with previous studies that found approximately 20–50% of subjects altered plans or were incapacitated due to TD, 10–20% being confined to accommodations, and 1% requiring hospitalization [37, 104–106]. While only 3% of TD cases were admitted for treatment, the high incidence of disease among long term travelers suggests that in situations such as large scale deployment of troops, severe, debilitating disease can be a large medical threat to operational readiness.

While the incidence of diarrheal disease and its associated morbidity among long term travelers does not appear to have changed since the original systematic review, there has been some improvement in the rates of care seeking. Compared to reported care seeking behavior described in 2006, subjects since that time have been roughly twice as likely to seek care for an acute gastrointestinal illness. It is uncertain what might explain this change, whether due to study differences or actual increasing awareness by service members of the potential effectiveness of therapies available over the course of a prolonged operational effort. Presenting to care early may lead to mitigation of morbidity from more severe cases and could potentially decrease the risk of developing post-infectious sequelae, such as irritable bowel syndrome, which is estimated to occur in up to 10% of cases. Observational studies show that illness duration and severity are independently associated with increased risk of PI-IBS [107]. Despite the finding that care-seeking behavior is improving, and at higher rates than reported in prior studies (5–15%), the overall rate of presenting to medical attention remains sub-optimal, at only about one in three cases, and remains a target for improvement [4, 98, 105, 106]. From a systematic standpoint, a recent cost effectiveness analysis of models to improve care seeking behaviors and optimize treatment among deployed military personnel demonstrated that modest cost increases could result in significant decreases in duty days lost to TD [108]. If applied across the military and civilian sector, this analysis demonstrates that time lost to illness could be decreased, and productivity of travelers increased, by focusing on encouraging sick individuals to seek care and optimizing treatment by providers.

This review includes a comprehensive literature search, prospective inclusion and exclusion criteria, standardized data abstraction, and current analytic methods, all of which reduce the potential bias in the resultant population of studies used for analysis. Limitations of this study include the heterogeneity of prevalence and incidence across the different included study designs, populations included, regions of study, and sparseness of data for some measures, particularly pathogen-specific outcomes of disease. While a small number of independent variables were found to explain some of the heterogeneity among included studies, small study numbers precluded further analysis of what factors may be associated with differential pathogen prevalence and disease incidence. Because several of the included studies in this review were performed in serial assessment from the same specific location within a region (e.g., military exercises in Thailand, student populations in Mexico), caution should be taken in generalizing these findings across an entire region. In addition the study excluded individuals traveling for business, leisure, or to visit friends and relatives (VFR), so our findings may not be applicable to these populations of travelers, though prior evaluation of excluded studies based on population non-eligibility did not find any appreciable differences in estimates of disease [109–113]. Our findings also highlight that there are significant differences in the etiology of diarrheal disease between deployed US military personnel and other long-term travelers, so collapsing the findings may not reflect the true epidemiology of disease in each population; however, as previously stated this finding may simply represent differing travel destinations and not an inherent difference in populations. The paucity of studies reporting epidemiologic data from important regions such as India, China, Oceana, and Sub-Saharan Africa is a major limitation of our systematic review. Lastly, this review focused primarily on endemic (sporadic) diarrheal illness occurring in long-term travel populations. Because pathogens associated with epidemic diarrheal illness also cause significant morbidity and can impact military operations, epidemiologic data on epidemic disease would be helpful, but is outside the scope of this review.

## Conclusion

This updated systematic review of studies on diarrheal illness among US military personnel and similar long-term travelers provides some important conclusions. Diarrheal disease among long-term travelers remains a frequent occurrence, and the associated morbidity is significant. While care-seeking behaviors have been improving over the last ten years, a high percentage of cases of diarrheal disease are not brought to medical attention. While it is unclear if these missed cases represent milder illnesses that are managed with self-treatment, capturing these cases could potentially result in decreased time spent incapacitated by illness or associated morbidity. Diarrheagenic *E. coli* (ETEC and EAEC, particularly), *Campylobacter*, and *Shigella* species remain significant diarrheal pathogens globally, with some variations in prevalence geographically. Among long-term travelers, a number of other bacterial, viral, and parasitic pathogens, though not encountered as frequently, need to be considered in cases of diarrheal disease. Lastly, because diarrheal disease remains frequent in this population, the high incidence of cases not seeking care remains high, and the associated morbidity of disease can be significant, diarrheal disease should be considered an important health threat and be investigated further with further well-designed studies, including those focused on timely and effective treatment strategies and preventative measures including behavioral or hygiene interventions and vaccine development.

## Additional file

Additional file 1: Systematic review of travelers’ diarrhea--data abstraction form. (DOC 69 kb)
