# Supplementary material for: Travelers’ diarrhea: update on the incidence, etiology and risk in military and similar populations – 1990-2005 versus 2005–2015, does a decade make a difference?
Source: Trop Dis Travel Med Vaccines. 2019 Jan 15;5:1. doi: 10.1186/s40794-018-0077-1 (PMC6332902; doi:10.1186/s40794-018-0077-1)
Supplement: Supplementary file 1 — Systematic review of travelers’ diarrhea--data abstraction form. (DOC 69 kb) [file 40794_2018_77_MOESM1_ESM.doc]

**SYSTEMATIC REVIEW OF TRAVELERS’ DIARRHEA--DATA ABSTRACTION FORM**

(N/A = Not applicable, NR = Not reported)

Lead Author: _____________________ Year: _______ Publication Name: _____________________________________________

Article Title (Short): _________________________________________________________________________________________

Publication Type:  Published article  Technical Report  Other____________________________

Study Design:  Descriptive  Cross-sectional  Case-control

Cohort  Clinical trial  Other_________________________­___­­­­­­­­­­

Country Name: ____________________ Geographic region: Latin Amer./Carib. SE Asia Middle East Africa Other

Study Population:  U.S. military  Non-U.S. military  Peace Corps  Student  Other ______________

Population size (N): _____ Duration of Travel (months):______ Med./Mean (circle one) Standard TD definition used:  Yes  No

Population Gender:_____%Male Age(yr):_____ Median/Mean (circle one) Years of Study Period _________-- _________

If military 🡪 Branch of Service: Army Navy Air Force Marines  Other____________________

🡪 Operational setting: Routine deployment  Exercise Combat  Other__________________

🡪 Rank: Enlisted:____% Officer:____%

The reference provides complete information for the following questions (check all that apply):

Question 1: What is the all diarrhea and pathogen-specific incidence of travelers’ diarrhea for ETEC, Campylobacter and Shigella?

Question 2: What are the probabilities that an individual will seek or not seek treatment?

Question 3: What are probabilities of different treatment modalities, adverse events, hospitalization, treatment failure found in cases that sought and did not seek treatment?

Question 4: What are the disease outcomes associated with treated and un-treated disease?

**Eligibility determination: (all must be checked)**

- Observational studies or clinical trials.
- Conducted in U.S. military or other similar traveler population.
- Geographic regions of Central America, SE Asia, Africa and the Middle East for deployments of any length of time.

**QUESTION 1: All Diarrhea & Pathogen Specific Disease Incidence**

**N/A 🡪 go to next question**

Diagnosis:  Culture  Indirect (PCR/EIA)  Serology  Other ___________________________

Prevalence/Incidence:

| PATHOGEN | Type of Measure | % Prevalence /  # of events | Denominator (person-time) | Attack Risk / Incidence estimate |
| --- | --- | --- | --- | --- |
| ETEC | AR  Prev  Incidence |  |  |  |
| Campylobacter | AR  Prev  Incidence |  |  |  |
| Shigella | AR  Prev  Incidence |  |  |  |
| Salmonella | AR  Prev  Incidence |  |  |  |
| Virus(1) ______________________ | AR  Prev  Incidence |  |  |  |
| Virus(2) ______________________ | AR  Prev  Incidence |  |  |  |
| Other(1)______________________ | AR  Prev  Incidence |  |  |  |
| Multiple pathogens | AR  Prev  Incidence |  |  |  |
| No pathogen isolated | AR  Prev  Incidence |  |  |  |
| All diarrhea | AR  Prev  Incidence |  |  |  |

**QUESTION 2: What are the probabilities that an individual will seek or not seek treatment?**

**N/A 🡪 go to next question**

Seek treatment measure: ______ per ______—_______ Based-on:  MTF visit  Self-report

events person time

No seek Treatment measure: ______ per ______—_______ Based-on:  Self-report  Other ____________

events person time

**Question 3: What are probabilities of different treatment modalities, adverse events, hospitalization, treatment failure found in cases who sought and did not seek treatment?**

**N/A 🡪 go to next question**

**P [moderate to severe side-effect (requiring additional treatment)]**:  **NR**

Adverse event (1)____________________________________ Probability _________ / __________ = __________%

Adverse event (2)____________________________________ Probability _________ / __________ = __________%

**P [moderate to severe complication due to illness (requiring additional treatment)]:**  **NR**

Complication event (1)________________________________ Probability _________ / __________ = __________%

Complication event (2)________________________________ Probability _________ / __________ = __________%

**P [hospitalization due to Tx of side-effect]**: **NR** Probability _________ / __________ = __________%

**P [hospitalization due to diarrhea]**: **NR** Probability _________ / __________ = __________%

**P [treatment failure]:** (Defn:_____________________________ Probability _________ / __________ = __________%

**P [treatment modalities]:**  **NR**

Sought Treatment  Require IV fluids------------🡪 Probability _________ / __________ = __________%

Antibiotics-------------------🡪 Probability _________ / __________ = __________%

Antimotility agent-----------🡪 Probability _________ / __________ = __________%

Require SIQ/Bedrest--------🡪 Probability _________ / __________ = __________%

Other_________________ 🡪 Probability _________ / __________ = __________%

Did not seek Treatment  Antibiotics--------------------🡪 Probability _________ / __________ = __________%

Antimotility agent -----------🡪 Probability _________ / __________ = __________%

Require SIQ/Bedrest--------🡪 Probability _________ / __________ = __________%

Other__________________🡪 Probability _________ / __________ = __________%

**Question 4: What are the outcomes (morbidity) associated with treated and un-treated disease?**

**N/A 🡪 done**

Sought Treatment Did not seek treatment or untreated (placebo)

Days diarrhea prior to Rx----🡪 _______ days  Total Duration diarrhea ----------------🡪 _______ days

Days diarrhea post to Rx----🡪 _______ days  Days of Sick in Quarters---------------🡪 _______ days

Time to last unformed stool-🡪 _______ days  Days Decreased performance---------🡪 _______ days

Days of Sick in Quarters ----🡪 _______ days  Other(1)_______________________🡪 _______ days

Days hospitalized ------------🡪 _______ days  Other(2)_______________________🡪 _______ days

Days decreased performance🡪 _______ days COMMENTS:_____________________________________________

Other(1)________________🡪 _______ days _______________________________________________________

Other(2)________________🡪 _______ days _______________________________________________________
